# Resolving the Sources of Plasma Glucose Excursions following a Glucose Tolerance Test in the Rat with Deuterated Water and [U-^13^C]Glucose

**DOI:** 10.1371/journal.pone.0034042

**Published:** 2012-03-30

**Authors:** Teresa C. Delgado, Cristina Barosa, Patrícia M. Nunes, Sebastián Cerdán, Carlos F. G. C. Geraldes, John G. Jones

**Affiliations:** 1 Intermediary Metabolism Group and Inorganic Biochemistry and Molecular Imaging Group, Center for Neurosciences and Cell Biology, Coimbra, Portugal; 2 Laboratory for Imaging and Spectroscopy by Magnetic Resonance, Instituto de Investigaciones Biomédicas de Madrid Alberto Sols CSIC/UAM, Madrid, Spain; Facultad de Medicina - Universidad Autonoma Madrid, Spain

## Abstract

Sources of plasma glucose excursions (PGE) following a glucose tolerance test enriched with [U-^13^C]glucose and deuterated water were directly resolved by ^13^C and ^2^H Nuclear Magnetic Resonance spectroscopy analysis of plasma glucose and water enrichments in rat. Plasma water ^2^H-enrichment attained isotopic steady-state within 2–4 minutes following the load. The fraction of PGE derived from endogenous sources was determined from the ratio of plasma glucose position 2 and plasma water ^2^H-enrichments. The fractional gluconeogenic contributions to PGE were obtained from plasma glucose positions 2 and 5 ^2^H-positional enrichment ratios and load contributions were estimated from plasma [U-^13^C]glucose enrichments. At 15 minutes, the load contributed 26±5% of PGE while 14±2% originated from gluconeogenesis in healthy control rats. Between 15 and 120 minutes, the load contribution fell whereas the gluconeogenic contribution remained constant. High-fat fed animals had significant higher 120-minute blood glucose (173±6 mg/dL *vs.* 139±10 mg/dL, *p*<0.05) and gluconeogenic contributions to PGE (59±5 mg/dL *vs.* 38±3 mg/dL, *p*<0.01) relative to standard chow-fed controls. In summary, the endogenous and load components of PGE can be resolved during a glucose tolerance test and these measurements revealed that plasma glucose synthesis *via* gluconeogenesis remained active during the period immediately following a glucose load. In rats that were placed on high-fat diet, the development of glucose intolerance was associated with a significantly higher gluconeogenic contribution to plasma glucose levels after the load.

## Introduction

Plasma glucose levels are controlled within a narrow range over the daily feeding/fasting cycle through a tight coordination of plasma glucose appearance and disposal. In insulin resistant and diabetic states, glycemic control is compromised and plasma glucose levels fluctuate much more widely, particularly after a meal or a glucose load. Over time, these plasma glucose excursions (PGE) mediate a wide range of harmful systemic effects that are the hallmarks of diabetes. The glucose tolerance test is a widely used procedure for evaluating the efficacy of glycemic control in both humans and animal models of insulin resistance and diabetes. Following a glucose load, the increased levels of plasma glucose promote pancreatic β-cells to secrete insulin, which induces peripheral, and splanchnic glucose uptake, while at the same time suppressing endogenous glucose production (EGP) from hepatic gluconeogenesis and glycogenolysis [1]. Since insulin-mediated suppression of EGP is a key component of the homeostatic response to a glucose challenge, resolving the endogenous and load contributions to PGE is of high interest in characterizing the loss of glucose homeostasis in insulin resistant and diabetic states.

**Figure 1:**
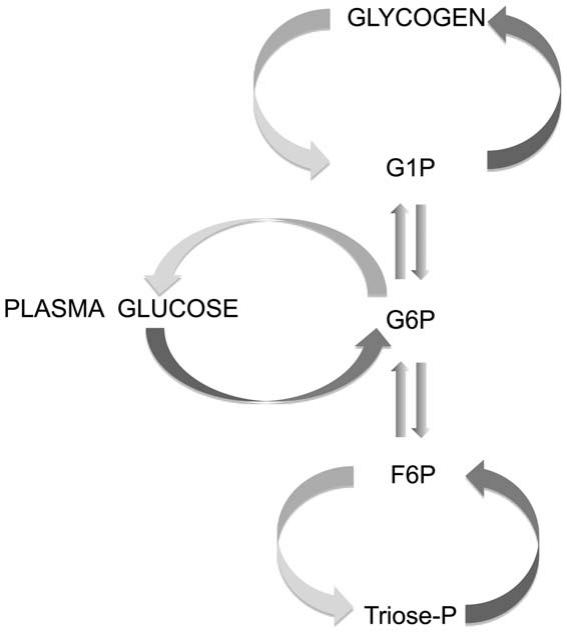
Metabolic fluxes connecting hepatic glucose-6-phosphate (G6P) with plasma glucose and with other hepatic metabolites: fructose-6-phosphate (F6P), glucose-1-phosphate (G1P), triose phosphates (Triose-P) and glycogen.

To date, the principal approach used to resolve endogenous and load contributions to PGE is based on measuring appearance rates of plasma glucose from all sources and from the glucose load through isotope dilution measurements of glucose tracers administered systemically and in the load. The endogenous contribution to PGE is estimated from the difference in systemic and load glucose rates of appearance [2], [3]. Total and load glucose rates of appearance are obtained by periodic measurement of plasma glucose enrichments or specific activities from both tracers and applying the data to compartmental [2], [4], [5] or circulatory models [6] that represent endogenous glucose pools and net glucose input and output rates. These analyses are subject to uncertainties associated with tracer appearance and exchange between endogenous glucose pools under isotopic and metabolic nonsteady-state conditions. An alternative approach is to directly label the immediate precursor of endogenous glucose, glucose-6-phosphate (G6P), and measure the appearance of this label in plasma glucose. This requires that the G6P precursor is instantaneously labeled to isotopic steady-state on administration of the glucose load. Moreover, the tracer has to represent all hepatic G6P molecules derived from all sources. To this end, we examined the possibility of administering deuterated water (^2^H_2_O) as part of an intraperitoneal (i.p.) glucose load. It is well known that in the presence of ^2^H-enriched plasma water (PW), the position 2 hydrogen of hepatic G6P becomes rapidly enriched with ^2^H due to exchange between G6P and fructose-6-phosphate (F6P), as indicated in Figure 1. G6P that is generated by gluconeogenesis is additionally enriched in position 5, due to exchanges at the level of the triose phosphates, and the solvent-metabolite exchange processes that confer this positional enrichment are also rapid. These processes are unlikely to be the rate limiting steps in attainment of steady-state hepatic G6P enrichment from a ^2^H_2_O bolus, but rather the time it takes for the bolus ^2^H to fully mix with PW and establish a constant PW precursor enrichment level.

In this report, we demonstrate that in rat this mixing interval is brief (∼2–4 minutes) and that plasma glucose is extensively enriched with ^2^H in positions 2 and 5 within 15 minutes of an i.p. glucose load enriched with ^2^H_2_O. This enrichment information allowed a direct assessment of endogenous and gluconeogenic contributions to PGE during its early dynamic phase. Thus, by supplementing ^2^H_2_O with [U-^13^C]glucose in the load and quantifying plasma glucose ^2^H and ^13^C-enrichment distributions by ^2^H and ^13^C Nuclear Magnetic Resonance (NMR) spectroscopy, both endogenous and load contributions to PGE following a glucose tolerance test were resolved. Our results show that following a glucose load gluconeogenesis is not altered in healthy rats whereas the glucose intolerance induced by high-fat feeding in otherwise healthy rats is driven by elevated contributions of gluconeogenesis to plasma glucose.

## Materials and Methods

### Materials

[U-^13^C]glucose (99% enriched) and deuterated acetonitrile (99.8% enriched) were obtained from Cambridge Isotopes Laboratories Inc. (Andover, MA, USA) and 70% ^2^H_2_O was obtained from Eurisotop (Saint Aubin, France). Other common reagents were purchased from Sigma-Aldrich (St. Louis, MO, USA).

### Ethics Statement

Animal studies were carried out in compliance with NIH Principles of laboratory Animal care (NIH Publications 85-23, revised 1985) and with the Laboratory Animals Care Guidelines of European Union (86/609/CEE). The Center for Neurosciences and Cell Biology of the University of Coimbra institutional ethics committee ‘specifically approved this study’ (Approval ID: 05/2005).

### Animal protocols

Adult male Sprague-Dawley rats were housed in a room on a 12-h light-dark cycle under constant temperature (22–25°C) and with *ad libitum* access to food (standard chow diet: 2.7% fat, 60% carbohydrate and 16% protein) and water. Another group of animals (324±20 g) was maintained for 20 days on a high fat diet (HFD) (45% of calories from fat, 35% from carbohydrate and 20% derived from protein, E15744-34, SSNIFF from Specialdiäten GmbHH®) and compared to body weight matched control animals (318±13 g) at 120 min after the glucose load. After overnight fed *ad libitum*, at 6 hours fast, rats were injected i.p. with a glucose load (1.5 mg glucose/g body weight) enriched with 9.6% [U-^13^C]glucose and dissolved in 4 mL of 70% ^2^H_2_O-saline. At various intervals after the glucose load, animals were sacrificed under heavy anesthesia and blood was collected for NMR Spectroscopy analysis of ^13^C and ^2^H-positional enrichments of blood glucose. Blood glucose was assessed using a standard glucometer and plasma samples were also separated for PW ^2^H-enrichment analysis [7].

### Plasma Glucose Processing

Blood was immediately deproteinized with a final volume of 4% perchloric acid and centrifuged for 15 min at 13,000 g and 4°C. The supernatant was neutralized with concentrated KOH, desalted by anionic-cationic exchange chromatography and evaporated to dryness. The conversion of plasma glucose to monoacetone glucose (MAG) was performed according to the literature [8]. Briefly, a mixture of 0.5 ml acetone/ml original plasma and concentrated anhydrous H_2_SO_4_ (40 µl/ml acetone) was added to the sample and stirred vigorously during 4 h. Ten ml of H_2_O were added and the pH adjusted to 2.2–2.3 followed by incubation at 40°C for 5 h. The samples pH was adjusted to ∼8.0 and then evaporated to dryness. The dry residue was further extracted using ethyl acetate and MAG samples were then stored at room temperature until NMR analysis. Noteworthy, MAG molecule reflects plasma glucose labeling patterns but with fully resolved ^2^H and ^13^C NMR signals.

### Nuclear Magnetic Resonance (NMR) Spectroscopy

**^13^C NMR Spectroscopy:** MAG samples were dissolved in deuterated acetonitrile for ^13^C NMR analysis. Proton-decoupled ^13^C NMR spectra were obtained with a Varian Unity 11.75 T system (Varian Instruments, Palo Alto, CA) equipped with a 5-mm broadband probe. Spectra were acquired at 25°C using a 90° pulse and a 2.5 s acquisition time. Typically 4,000–10,000 scans were averaged.

**^2^H NMR Spectroscopy:** MAG proton-decoupled ^2^H NMR spectra were obtained using a Varian 14.10 Tesla (T) spectrometer (Varian Instruments, Palo Alto, CA) equipped with a 3-mm broadband probe. MAG was dissolved in 0.2 ml 90% acetonitrile/10% water and shimming was performed on selected ^1^H resonances of MAG. Proton-decoupled ^2^H NMR spectra were acquired at 50°C using a 90° pulse and a 1.5 s acquisition time. Typically 6,000–20,000 scans were averaged. For the determination of PW ^2^H-enrichment from ^2^H_2_O using the method described in Jones *et al.* [7], ^2^H NMR spectra were obtained at 25°C using a 22.5° pulse, an 8 s pulse delay, and a 4 s acquisition time.

**NMR Analysis:** ^13^C and ^2^H NMR spectra were analyzed using the curve-fitting routine supplied with the NUTS PC-based NMR spectral analysis program (Acorn NMR Inc., Fremont CA). ^13^C NMR excess enrichments were determined relative to the natural abundance C1 singlet (1.11%) whereas ^2^H NMR enrichments were quantified relative to an internal standard of dimethylsulfoxide (DMSO).

### Plasma glucose sources

Plasma glucose sources were resolved into load and endogenous contributions by using [U-^13^C]glucose and ^2^H_2_O tracers administered as part of the load. The contribution of the i.p. glucose load to total plasma glucose concentration was estimated using Eq.[1]. Plasma and load [U-^13^C]glucose enrichments were quantified by ^13^C NMR analysis of the MAG derivative as previously described [9], [10], [11], [12]:
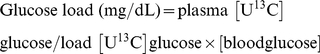
(1)The ^13^C-label of [U-^13^C]glucose may be recycled into glucose *via* the Cori cycle (glucose→lactate→glucose). The principal glucose isotopomers produced by this pathway are partially labeled ([1,2,3-^13^C_3_]glucose+[1,2-^13^C_2_]glucose) and can be distinguished from the parent [U-^13^C]glucose isotpomer in the ^13^C NMR spectrum [9], [13]. The abundance of these isotopomers, corrected for dilution at the level of the tricarboxylic acid (TCA) cycle, provides an estimate of the fraction of plasma glucose derived from recycling of the [U-^13^C]glucose load [9], [13] as follows:
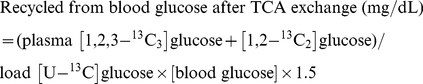
(2)Where 1.5 is a correction factor that accounts for dilution of the ^13^C-label at the level of the hepatic TCA cycle [9].

In addition to absorption from the load, plasma glucose is also generated endogenously by EGP and this process results in enrichment from ^2^H_2_O. All glucose molecules that were derived from endogenous G6P are enriched in position 2, hence the fraction of plasma glucose molecules derived endogenously is equal to the ratio of glucose position 2 (glucose H2) and PW precursor ^2^H-enrichments, quantified by ^2^H NMR Spectroscopy, and is expressed as the concentration component of blood glucose by Eq.[3]:
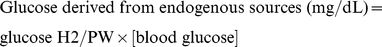
(3)Noteworthy, the endogenous contribution will include glucose molecules that participated in glucose-G6P cycling since they are also enriched in position 2.

Endogenous glucose that was produced *via* gluconeogenic pathways is enriched in position 5 as well as position 2 due to additional exchanges at the level of the triose phosphates, whereas glucose derived from either glycogenolysis or glucose-G6P cycling is not enriched in position 5 [12]. Hence, the gluconeogenic contribution to plasma glucose can be estimated from the ratio of plasma glucose position 5 enrichment (glucose H5) and PW and expressed as the concentration component of blood glucose according to Eq.[4]:
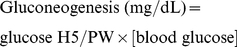
(4)


### Statistical Analysis

Data are expressed as means ± SEM. Statistical differences with *p*<0.05 were determined using Student's *t*-test, assuming that data showed a normal distribution.

## Results

### Plasma glucose excursions (PGE) and sources

**Figure 2:**
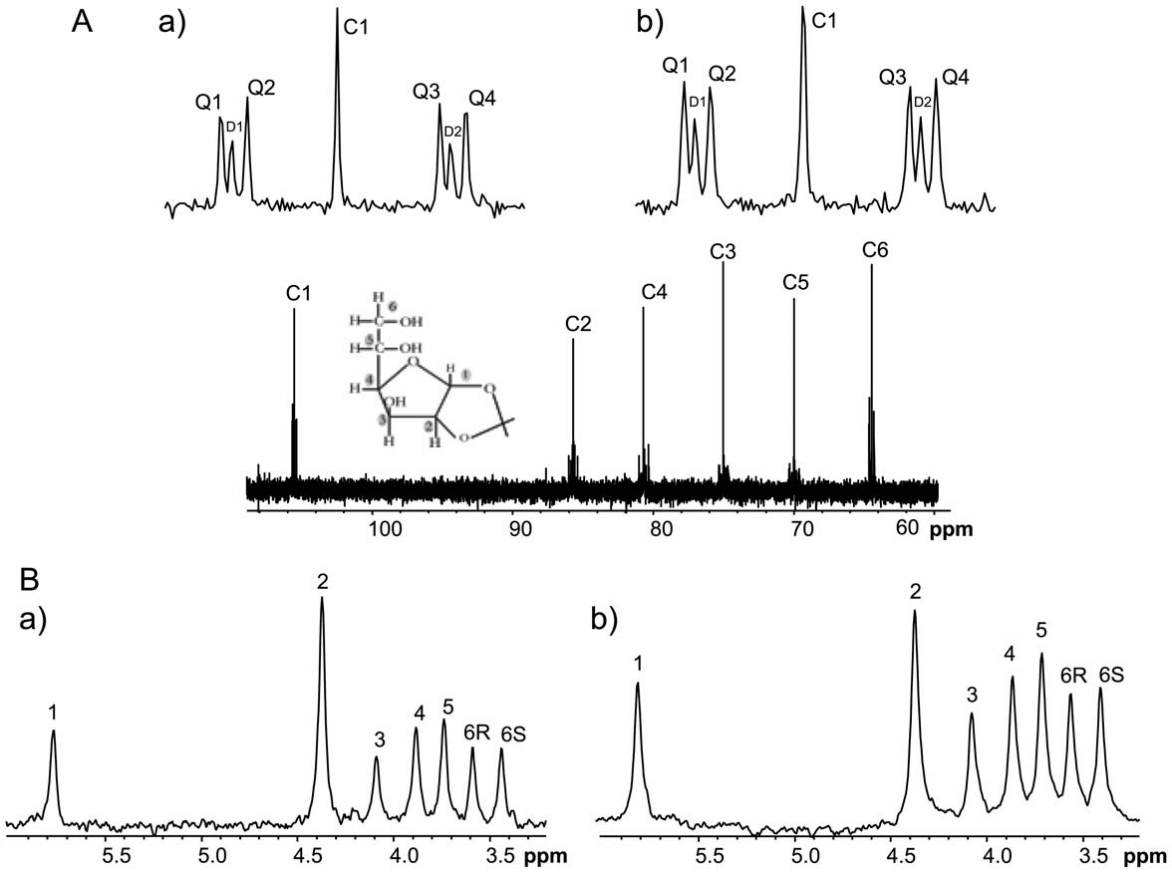
^13^C NMR spectrum of monoacetone glucose (MAG) derived from plasma glucose 120 minutes after an intra-peritoneal glucose load enriched with [U-^13^C]glucose (1.5 mg/g body weight) (A) in a) healthy control and b) HFD fed-animals. The singlet (C1) corresponding to the natural abundance and [U-^13^C]glucose isotopomer (the quartet Q1–Q4) are represented in the inset. The doublet signals (D1 and D2) are due to ^13^C-^13^C coupling between carbons 1 and 2 (but not carbon 5) and represent the sum of [1,2,3-^13^C3]glucose and [1,2-^13^C2]glucose isotopomers. ^2^H NMR spectrum of MAG from pooled samples (B) from a) control and b) HFD-fed animals 120 min after an intraperitonral glucose load (1.5 mg/g body weight) enriched with deuterated water (^2^H_2_O). Seven MAG aliphatic hydrogens derived from plasma glucose are shown and identified respectively.

**Figure 3:**
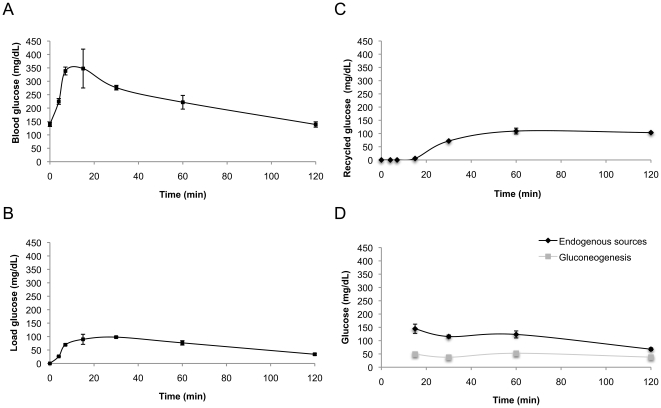
Source contributions to plasma glucose excursions (PGE) following an intra-peritoneal glucose load enriched with [U-^13^C]glucose and deuterated water (^2^H_2_O). A. blood glucose profile; B. Plasma glucose concentration attributable to the load glucose; C. Plasma glucose concentration attributable to Cori cycle activity and D. Plasma glucose concentration attributable to total endogenous sources and to gluconeogenesis. Data are represented as average ± standard error means of at least n = 3 *per* time point.

The contribution of the i.p. glucose load to PGE was estimated from plasma [U-^13^C]glucose enrichment levels as previously described [9], [10], [11], [12] and a representative ^13^C NMR spectrum of plasma glucose sampled at 120 min from a control rat is shown in Figure 2A a). In control healthy rats, blood glucose peaked at 15 min after the glucose load and decreased gradually thereafter, returning to pre-load levels after 120 min (Figure 3A). While total blood glucose levels crested at above 300 mg/dL at 15 minutes post-load, the load glucose only accounted for about one third of this excursion (Figure 3B), indicating that the initial surge in plasma glucose levels following the glucose tolerance test is only partly accounted for by absorption of the glucose load. Thereafter, the absolute contribution of the load glucose to PGE decreased slowly. Recycled glucose isotopomers from Cori cycle activity were undetectable until about 15 minutes after the glucose load. From 15 to 60 minutes after the glucose load, the amount of recycled glucose rose gradually and then remained constant up to 120 minutes (Figure 3C). Recycled glucose contributions to PGE increased over time from 2% at 15 minutes to 75% at 120 minutes.

Following the i.p. glucose and ^2^H_2_O load, plasma water ^2^H-enrichment attained constant levels within 2–4 minutes, as shown in Table 1. The early establishment of constant PW ^2^H-enrichment levels provides the basis for estimating the fractional endogenous and gluconeogenic contributions to PGE early after administering the glucose load. ^2^H-positional enrichments of plasma glucose were analyzed by ^2^H NMR Spectroscopy analysis of the MAG derivative. To ensure a high level of certainty for glucose ^2^H-enrichment measurements for each time point and with the initial expectation that plasma glucose ^2^H-enrichment following a glucose load would be relatively low, MAG samples from several animals were pooled in order to obtain ^2^H NMR spectra with high signal-to-noise ratios [Figure 2B a)]. Enrichment of glucose position 2 relative to PW informs the fractional contribution of total endogenous sources to PGE while enrichment of glucose position 5 relative to PW informs the contribution of *de novo* synthesized glucose (i.e. gluconeogenesis) [12].

**Table 1:** Plasma water excess ^2^H-enrichments following an intraperitoneal glucose load enriched with deuterated water (^2^H_2_O).

Time (min)	Average ± standard error mean (%)^a^
0	n.d.^b^
1	0.81^c^
2	0.78±0.10
4	1.21±0.08
7	1.42±0.20
15	1.05±0.10
30	1.03±0.02
60	1.00±0.03
90	1.07±0.13
120	0.94±0.05

a at least n = 3 per time point.

b n.d. ^2^H-signal for water not detected (signal-to-noise <3.0).

c n = 1.

The earliest ^2^H NMR spectrum was obtained at 15 minutes after the load, corresponding to the crest of plasma glucose levels. At this time, the contribution of endogenous sources to the plasma glucose levels exceeded that of the glucose load (Figure 3D). The gluconeogenic contribution to PGE represented about one-third of total endogenous contributions and about 14% of the total glucose excursion. During the subsequent fall in plasma glucose levels between 15–120 minutes, the plasma glucose concentration attributable to gluconeogenesis remained remarkably constant at ∼50 mg/dL. Consequently, the fractional gluconeogenic contribution to PGE steadily rose reaching ∼30% at 120 minutes. Meanwhile, the total endogenous contribution to plasma glucose levels (which in addition to gluconeogenesis includes glucose derived from glycogenolysis and futile glucose-G6P cycling) was maximal at 15 minutes representing ∼145 mg/dL or ∼40% of the total plasma glucose. From 15–120 minutes, the plasma glucose concentration attributable to endogenous sources fell in step with that of total plasma glucose such that the fractional endogenous contribution to plasma glucose levels at 30, 60 and 120 minutes (around 40%, 57% and 50%, respectively) were similar to that at 15 minutes. In summary, the ^2^H-enrichment data demonstrate that gluconeogenic output was remarkably unaffected by the glucose load while total endogenous contributions slowly decreased from 15–120 minutes after the load.

### Profiling of glucose metabolism at a single time point after the glucose tolerance test

The proposed NMR method requires 1–2 ml of whole blood to obtain precise measurements of plasma glucose ^2^H-enrichment from ^2^H_2_O and ^13^C-enrichment from [U-^13^C]glucose and is therefore limited to a single endpoint measurement. To determine if the tracer information could be integrated with standard plasma glucose concentration measurements following a glucose load, we performed a set of studies where glucose sources at 120 minutes after a [U-^13^C]glucose and ^2^H_2_O load were measured by the tracer methods following conventional glucometer measurements of PGE. These studies were applied to control animals fed on a normal chow diet and a second group fed with a HFD - a well-known procedure for inducing insulin resistance and glucose intolerance associated with increased gluconeogenesis [14] [Figures 2 A b) and B b)].

**Figure 4:**
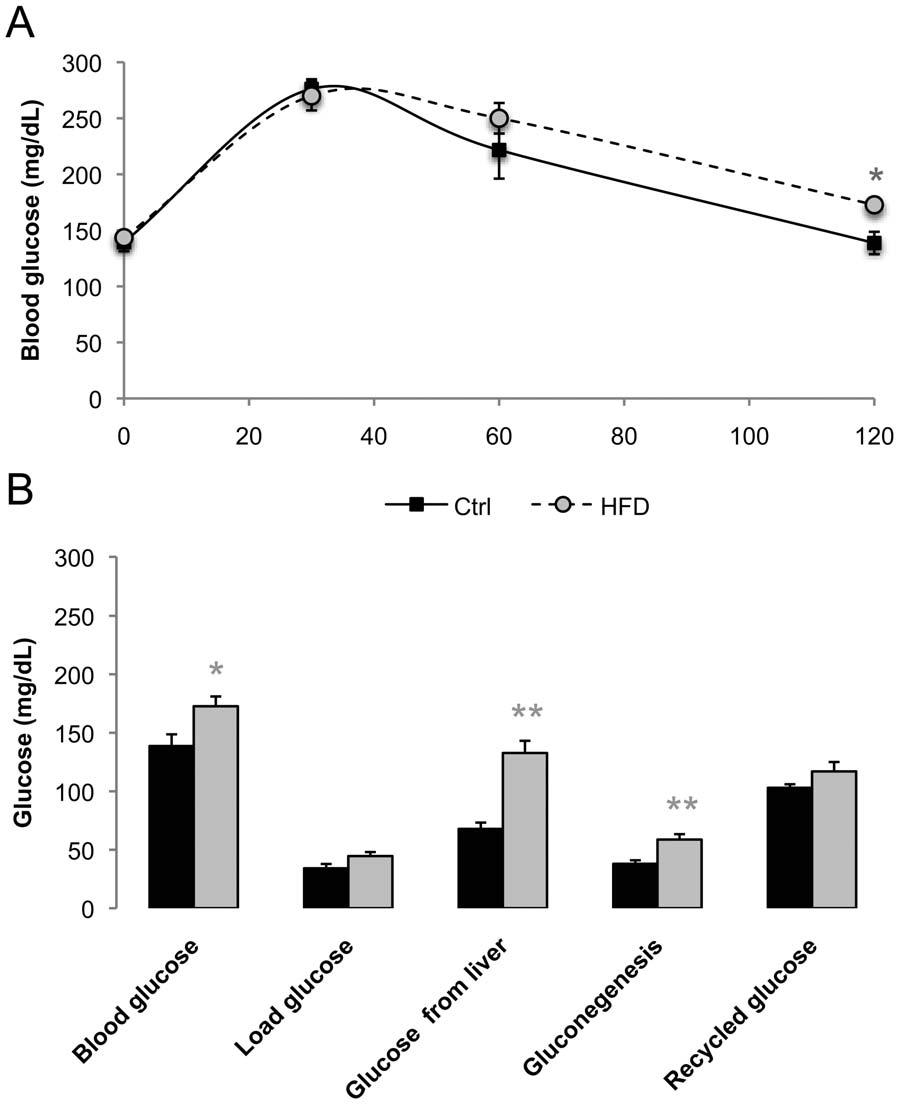
Plasma glucose excursions (PGE) during an i.p. glucose load enriched with [U-^13^C]glucose and ^2^H_2_O of control (Ctrl) rats fed on normal chow diet and a group maintained on a high fat diet (HFD) (A); and plasma glucose source contributions at 120 min after the load (B). Means and standard error means of individual animals (5 for Ctrl and 7 for HFD) are shown. *p<0.05 and **p<0.01 relative to controls is indicated.

Animals maintained on HFD showed normal fasting blood glucose levels, but were glucose intolerant and mildly insulin resistant as demonstrated by impaired clearance of the glucose load. The higher plasma glucose attained at 120 min after the labeled glucose load in the HFD animals was associated with significant alterations in the plasma glucose sources compared to rats fed on normal chow. While plasma glucose levels attributable to the [U-^13^C]glucose load were similar between the two groups (45±3 mg/dL in HF-diet rodents and 34±4 mg/dL in control animals), those derived from endogenous sources (both gluconeogenic and non-gluconeogenic) were significantly increased (133±11 mg/dL *versus* 68±5 mg/dL for total endogenous sources and 59±5 mg/dL *versus* 38±3 mg/dl for gluconeogenesis, *p*<0.01 relative to controls) (Figure 4 A and B). Recycled glucose from Cori cycle was not significantly increased in HFD rats relative to controls (117±8 mg/dL *versus* 103±3 mg/dL in control animals).

## Discussion

Direct measurements of load and endogenous glucose contributions to PGE following an i.p. glucose tolerance test were resolved in rats by using a glucose load enriched with [U-^13^C]glucose and ^2^H_2_O followed by NMR analysis. These studies revealed a surprisingly low contribution of load glucose to the initial increase in plasma glucose levels at 15 minutes after the load, whereas endogenous contributions, including both gluconeogenic and non-gluconeogenic sources, were found to be significant. Previous studies with oral glucose loads in 6-h fasted rats show that 60 min after the load, only 50% of plasma glucose was accounted for by the load [15] while in 24-h fasted rats, the load contribution was higher, representing 70–80% of the total plasma glucose concentration [12]. Thus, the reduced load glucose contribution most probably reflects a higher initial glucose pool size and/or increased basal EGP at 6-h *versus* 24-h fasting rather than a reduced absorption rate of the i.p. glucose load relative to an oral glucose load.

The endogenous contribution was based on the analysis of glucose ^2^H-enrichment from ^2^H_2_O and assumed a constant level of PW ^2^H-enrichment throughout the experiment. While establishment of PW ^2^H-enrichment after the load was rapid, it was not instantaneous. To the extent that hepatic glucose that was synthesized before ^2^H_2_O from the i.p. load became fully equilibrated with PW, the observed ^2^H-enrichment of plasma glucose could in part reflect the dynamics of ^2^H_2_O mixing. Indeed, the PW enrichment profile hints at an initial pulse of ^2^H-appearance with enrichments at 4 and 7 minutes being somewhat higher compared to later times. Assuming similar appearance of ^2^H_2_O in plasma and hepatocyte water, enrichment of glucose that was synthesized during this brief surge of ^2^H_2_O would be higher than expected resulting in overestimates of endogenous fractions. Meanwhile, glucose that was synthesized during the initial appearance of ^2^H_2_O between 0 and 4 minutes after the load would be less enriched. We believe it is unlikely that endogenous contributions to PGE are being substantially over-reported by the ^2^H_2_O method. Indeed, a high contribution of endogenous sources to plasma glucose levels is entirely consistent with our observation that the load glucose accounted for only a minority of PGE during the early stages, as seen by the low plasma [U-^13^C]glucose enrichment levels derived from the glucose load. If this approach is applied to larger animals and humans, where the interval for the ^2^H_2_O loading dose to become fully equilibrated with body water is likely to be longer [16], glucose ^2^H-enrichment at early times would need to be appropriately corrected for nonsteady-state body water ^2^H-enrichment levels.

The substantial endogenous contribution to total plasma glucose excursion, as determined from the enrichment of plasma glucose from ^2^H_2_O given with the glucose load, was unanticipated given that the expected response to a rapid increase in plasma glucose levels is the suppression of endogenous glucose release. Under normal feeding conditions, this suppression is mediated by increased portal vein insulin and glucose levels that promote net glucose conversion to G6P and glycogen, as well as a reduction in glucagon levels - a potent stimulator of both hepatic gluconeogenesis and glycogenolysis. Depending on the composition of the meal, glucagon levels may rise [17], [18]. In humans, the endocrine response to a glucose tolerance test differs from that of a normal meal in that fasting glucagon levels are maintained [19]. If the same is true for rats, this would explain the persistent endogenous contribution to PGE during the glucose tolerance test despite the increased insulin levels. Moreover, the persistence of gluconeogenic activity following the glucose load is in agreement with previous studies showing that gluconeogenic flux is relatively insensitive to acute increases in insulin and glucose [20], [21], [22].

In 6-h fasted control rats, EGP was reported to be inhibited by 30% at 60 minutes after an oral glucose load [15], whereas in 24-h fasted rats EGP was not suppressed [23], [24]. To the extent that EGP as measured by plasma glucose ^2^H-enrichment of position 2 includes contributions from glucose-G6P futile cycling, the true rates of EGP (i.e. hepatic glycogenolysis plus gluconeogenesis) may be overestimated. In hepatocytes, it was shown that futile cycling between glucose and G6P is extensive when glycogen content is elevated (i.e., fed animals) and relatively low in cells from starved rats [25]. Considering that 6-h fasted animals demonstrated substantial G6P-ase activity (as seen by the substantial contribution of EGP to whole body glucose) in the face of a large glucose load and increased insulin levels (which could stimulate glucokinase), glucose-G6P cycling could be active under these conditions and would therefore contribute substantially to the fraction of EGP assigned to glycogenolytic flux.

The HFD intervention resulted in elevated endogenous contributions to PGE indicating that our measurement was sensitive to diet-induced changes in hepatic carbohydrate metabolism. Our approach directly reveals that the increased endogenous contribution was fuelled by gluconeogenic activity and is also consistent with the development of hepatic insulin resistance following high fat feeding [26]. These alterations attributable to HFD are consistent with a previous rodent study where high fat feeding was shown to induce hepatic insulin resistance as assessed by the hyperinsulinemic-euglycemic clamp [14]. Recycled glucose from Cori cycle is not significantly increased in HFD rats relative to control animals suggesting that other substrates such as amino acids and glycerol are being used as sources of gluconeogenesis in HFD rodents.

Resolution of endogenous and load contributions to PGE by [U-^13^C]glucose and ^2^H_2_O informs the response of hepatic glucose metabolism to insulin and hepatic insulin resistance. The current gold-standard method for evaluating hepatic insulin resistance is the hyperinsulinemic-euglycemic clamp. Compared to the clamp measurement, our approach is considerably simpler since the surgical and analytical maneuvers for maintaining constant plasma glucose and insulin levels are not present. The principal theoretical limitation of our method is that in the absence of an infused glucose tracer to determine plasma glucose turnover, absolute rates of glucose appearance from endogenous and load sources cannot be directly calculated from plasma glucose levels and tracer enrichments. Another key practical limitation of the method at the present time is the relatively large volume of blood (∼2 ml) needed for performing ^2^H and ^13^C NMR analyses, which precludes multiple samplings from a single rat. While ^13^C sensitivity could be improved by indirect-detection techniques [27], equivalent methods for improving the sensitivity of ^2^H analysis - while retaining the quantitative accuracy and precision of direct ^2^H-detection - have not yet been developed. In our study, plasma samples from several animals were pooled solely to maximize the signal-to-noise ratio of the glucose ^2^H NMR signals. There are several factors that could substantially improve sensitivity so that glucose ^2^H-enrichment from single blood samples may be precisely quantified. Firstly, PW enrichment levels of 3% can be established with a single loading bolus in rats, [28], [29] and this modification alone would provide a 3-fold increase in ^2^H-precursor enrichment levels over that of the present study. Cold-probe technology offers a 3–4 fold improvement in sensitivity over conventional probes, further reducing the minimum sample size. With these modifications, we estimate that ∼0.5 ml whole blood would provide sufficient signal-to-noise for precise analyses of glucose ^2^H and ^13^C-enrichments. A 350 gram adult rat has a total blood volume of ∼22 ml [30] and 6–12% of this (∼1.3–2.6 ml) may be withdrawn from a conscious animal with minimal changes in hemodynamic and autonomic responses and blood gas levels [31]. This amount would permit 4–5 samples of 0.5 ml to be removed and would allow multiple determinations of plasma glucose sources over the glucose tolerance test period for a single animal. Alternatively, more sensitive methods such as Gas Chromatography-Mass Spectrometry (GC-MS) could be used to resolve enrichments from ^2^H_2_O and a labeled glucose load.

In summary, we demonstrated that both endogenous and load components of plasma glucose excursions can be resolved in a direct way during a glucose tolerance test by administering an i.p. glucose load enriched with ^2^H_2_O and [U-^13^C]glucose and resolving plasma glucose ^2^H and ^13^C-enrichment distributions by NMR Spectroscopy. This approach revealed that hepatic gluconeogenesis is not altered following a glucose load and that the development of glucose intolerance in healthy rats by high-fat feeding is driven in part by significant alterations in endogenous glucose production from gluconeogenesis.
